# Tobacco smoking, associated risk behaviours, and experience with quitting: a qualitative study with homeless smokers addicted to drugs and alcohol

**DOI:** 10.1186/1471-2458-13-951

**Published:** 2013-10-10

**Authors:** Laura Garner, Elena Ratschen

**Affiliations:** 1 Nottinghamshire Healthcare NHS Trust, Broad Street, Nottingham NG1 3AL, UK; 2 Department of Epidemiology and Public Health; City Hospital, Clinical Sciences Building, University of Nottingham, Hucknall Road, Nottingham NG5 1PB, UK

**Keywords:** Tobacco, Smoking, Smoking cessation, Homeless, Vulnerable groups

## Abstract

**Background:**

The prevalence of tobacco smoking among homeless people can reach more than 90%, with related morbidity and mortality being high. However, research in this area is scarce. This study aims to explore smoking and quitting related behaviours, experiences and knowledge in homeless smokers in the context of other substance abuse.

**Methods:**

Face-to-face interviews were conducted with homeless smokers accessing a harm reduction service in Nottingham, UK. Data on smoking history, nicotine dependence, motivation and confidence to quit were collected using structured instruments; a semi-structured interview guide was used to elicit responses to predefined subject areas, and to encourage the emergence of unprecedented themes. Data were analysed using framework analysis and descriptive statistics.

**Results:**

Participants were generally highly dependent smokers who did not display good knowledge/awareness of smoking related harms and reported to engage in high risk smoking behaviours. The majority reported notable motivation and confidence to quit in the future, despite or indeed for the benefit of addressing other dependencies. Of the many who had tried to quit in the past, all had done so on their own initiative, and several described a lack of support or active discouragement by practitioners to address smoking.

**Conclusion:**

High levels of tobacco dependence and engagement in unique smoking related risk behaviours and social interplays appear to add to the vulnerability of homeless smokers. Given reported motivation, confidence, previous attempts and lack of support to quit, opportunities to address smoking in one of the most disadvantaged groups are currently missed.

## Background

Smoking is the largest single avoidable cause of premature death, disease and disability in the developed world. Over 100,000 people in the UK die from their own smoking or from environmental tobacco smoke exposure every year, and with a direct annual cost of tobacco related morbidity of £5 billion to the National Health Service (NHS), the economic burden is enormous [1]. Half of all smokers, most of whom are socioeconomically and otherwise disadvantaged [2], will die prematurely from their smoking unless they quit [3].

Smoking has been identified as a major contributor to health inequalities, with smoking prevalence and rates of premature smoking related morbidity and mortality substantially raised among socioeconomically and otherwise disadvantaged groups in society [4]. While smoking prevalence has been steadily declining in the general population, to currently around 21% in the UK, no decline is to date detectable among some of the most vulnerable groups [5]. One of these groups is the homeless, where the prevalence of smoking has been found to reach up to 96% [6], with early onset of smoking and heavy dependence being the norm, and comorbid dependency on alcohol and other drugs well recognised [7,8].

Homelessness is associated with substantially increased morbidity and mortality, with the average age at death among those who remain homeless estimated at 40–44 years [9]. This high morbidity and mortality has been shown to be attributable to a spectrum of health problems including alcohol abuse, illicit drug use and mental illness; however it includes a substantial component of diseases caused directly by smoking, including ischaemic heart disease, lung and other cancers [10].

In the context of complex life circumstances that often involve mental disorder, substance abuse and dependence, smoking and smoking cessation are often overlooked in the homeless population [11]. Given the commonly poor engagement with general health services [9], access of free NHS Stop Smoking Services (SSS) available in the UK is likely to be rare. Although research in the area remains scarce, a small number of international studies indicate that homeless smokers can be motivated and able to address their smoking. This study aims to explore homeless smokers’ views, attitudes, experiences and knowledge with regard to smoking and quitting in an urban UK setting.

## Methods

### Study design

The study consisted of semi-structured face-to-face interviews conducted with homeless smokers in Nottingham, UK.

### Setting & participants

Interviews were conducted in a drug harm reduction and sexual health service commissioned by the NHS in Nottingham city centre. The setting was regularly frequented by members of the local homeless community for advice and support related to common substance abuse (e.g. needle exchange) and was the work place of the principal researcher, a harm reduction specialist nurse. Service users were eligible for inclusion in the study if they were over 18 years of age, current smokers, able to understand English and to give informed consent, and classed as currently homeless, with homelessness defined as involving ‘rooflessness’ (rough sleepers), ‘houselessness’ (those living in hostels and temporary accommodation) and ‘living in overcrowded and insecure buildings’ [12].

Homeless smokers were identified by service staff using clinical notes and verbal confirmation, and recruited using purposive and snowball sampling strategies, aiming for the inclusion of participants who were not intoxicated at the time of approach, a range of age groups, representation of both genders (acknowledging that homelessness is more prevalent among males [13]), and a size of the sample that would indicate data saturation [14]. Potential participants were invited to take part and provided with study information, read aloud to allow for common literacy issues. A 24 hour ‘cooling off’ period applied before arranging interviews with informed written consent. It was emphasized that not taking part would not impact future use of the service.

### Study instruments

A semi-structured interview guide was devised, covering structured questions on demographics, smoking history and nicotine dependence in a structured manner (including questions to score participants’ dependence using the Heaviness of Smoking Index, HSI [15], a combined measure of number of cigarettes smoked and time to first cigarette after waking), while loosely guiding the exploration of smoking and quitting related behaviours, experiences and attitudes to allow for the emergence of novel themes thereafter. Interviews were tape recorded and transcribed verbatim.

### Data analysis

Structured data were collated and descriptively summarised using Microsoft Excel. Narrative data were analysed using framework analysis [16], identifying predefined and emerging themes and subthemes from the raw data and coding data transcripts accordingly, using manual data management techniques, such as highlighting and cutting out of themed sections. Transcripts were read and re-read, and codes identified and refined by two researchers (the principal researcher and an academic researcher in a supporting role). The contents of the main themes are summarised with relevant verbatim quotes in the results section to illustrate findings.

Throughout the conduct of interviews, transcription and data analysis, the duality of the principal researcher’s role (researcher/interviewer and harm reduction service provider as specialist nurse) was taken into account by reflecting on potential dynamics of the social interplay that could have influenced participants’ accounts. However, the researcher maintained a non-judgemental, neutral, yet encouraging position and perceived her professional involvement with the population as a strength that improved accessibility to an extremely hard-to-reach group.

The study was approved by the University of Nottingham Research Ethics Committee, and the Nottingham NHS Research Ethics Committee 2.

## Results

A sample of 15 participants had been recruited and interviewed in January and February 2012, when both researchers, upon reading and re-reading transcripts, felt that the point of data saturation had been reached. This occurred after especial (and successful) efforts had been made to recruit more than the original two female homeless service user into the study. Participants’ demographics, homelessness status and other substance use are summarised in Table 1.

**Table 1:** Participant characteristics

**Gender**	
Male	11
Age range (mean)	18 – 53 (33)
Currently sleeping	
In a hostel or winter shelter	6
Rough (on streets)	5
Sofa surfing*	4
Concurrent (multiple) substance use	
Alcohol	8
Heroin	4
Crack	4
Cannabis	6
Amphetamine	3
Methadone	6
Currently receiving treatment for drug or alcohol misuse	9
Age started smoking	
<16	15
Median age started smoking	13
Average number of cigarettes smoked daily (range)	20 (6–50)

*The practice of moving from one acquaintance, friend or relative’s house to another, sleeping in whatever spare space is available, floor or sofa, for a night or up to a few days before moving on to the next house.

Most participants scored values greater than 4 (out of 6) on the HSI (15), with 11 participants indicating that they smoked within 5 minutes of waking and 12 participants consuming between 11 and 30 cigarettes per day. Four participants scored values between 1 and 3, and only one participant had a score of 0, indicating they waited 60 minutes until smoking and smoked less than 10 cigarettes a day.

In the thematic analysis, the following three main themes were identified for coding after organising the contents of the transcripts thematically:

•Perception of the physical and mental health impacts of smoking

•Sourcing of tobacco and risk behaviour

•Smoking, quitting and harm reduction: environmental influence, past experience and future needs

The contents of each theme are described in greater detail, and illustrated with quotes, in the subsequent paragraphs.

### Perception of the physical and mental health impacts of smoking

All participants expressed awareness that smoking was harmful; however, none were able to name more than two smoking related conditions. Cancer and lung problems were cited most frequently as smoking related illnesses, and impact on looks and physical fitness were also commonly mentioned. Smoking appeared to be regarded as a relatively minor risk in the context of generally challenging and risky life circumstances:

*‘Living is risky; it* [the risk] *is the environment you’re brought up in and the environment that you live in at the minute’* (15; male, 18)

Scepticism of the actual risks of smoking was expressed on several occasions, with some believing smoking was held unduly responsible for some harms, and the majority of participants feeling that it did not impact on their own health significantly, in some cases appearing to ignore or playing down likely associations with physical health problems:

Interviewer (on the subject of participant’s asthma): ‘Does anything help when you can’t breathe in the morning?’

Participant: ‘A fag!’

Interviewer: ‘Have you ever thought that smoking could make it worse?’

*Participant: ‘Yeah and no…I might wake up in the morning really chesty and use my inhalers, but my inhalers are sitting on top of my fag packet (laughs)’* (12; female, 35)

### Sourcing of tobacco and risk behaviour

The affordability of smoking and sourcing of tobacco was described by most as a matter of constant concern, with legal tobacco largely unaffordable and the acquisition of contraband products a regular occurrence. There was widespread acknowledgement that for less money, one had to expect lower quality of tobacco products as well.

*‘(…). You’re paying over the odds for that [legal tobacco] and sometimes you haven’t got the money for it, that’s why, depending on how much money you’ve got, depends on what quality you get’* (8; male, 37)

Accessing cheap sources of illegal products, the range of participants’ weekly spend on tobacco was £5-15, which was regarded as an acceptable amount to sustain the behaviour. Most participants voiced that without this cheap source, they would find other ways of obtaining tobacco.

*Participant: ‘[If I had to pay the full price for tobacco], I wouldn’t be able to find the money, so I’d just be in town picking dog ends up’* (11; male, 44)

*‘Well, I’d go to the shop and I’d rob something that I can go and exchange for a pouch of tobacco or whatever’* (12; female, 35)

Sharing cigarettes was a practice participants engaged in to acquire tobacco, and there was evidence of this practice being determined by an intricate set of individual ‘rules’ and preferences: For some, the relationship with the potential sharer was pivotal, particularly relating to whether they had the first half of the cigarettes (‘first twos’) or the second half (‘second twos’). The reasons given for preferring ‘first twos’ related to concerns around hygiene and fear of transmittable diseases. For people the participants were less close to, many expressed that they would only ever give away ‘second twos’, as being the cigarette’s owner gave them the right to decline smoking a cigarette that someone else had had contact with. The hierarchy of sharing cigarettes was acknowledged by a participant who would accept second twos on occasion;

*‘Yeah, if its mine, I’ll have first twos on it, but if it’s someone else’s, then you can’t argue. You’ve got standards, but you can also change when you’re skint’* (8; male, 37)

The majority of participants disclosed that they engaged currently or had engaged in the past in smoking behaviours that exceeded the normal risks of smoking, such as smoking tobacco previously discarded by others on pavements or public cigarette bins. All said they only smoked discarded tobacco when they had no other way of sourcing it, and that they removed tobacco from discarded cigarettes, as this was seen to make the act more acceptable.

*‘But I don’t smoke it through their filters, I just take the baccy out and put it into a rizla and put my own filter in and smoke it like that’* (9; male, 40)

*‘It’s very rare that I’ll do it because I don’t like doing it, it’s trampy, but needs must when you need a smoke.’* (13; male, 33)

The three interviewees that had never smoked discarded cigarettes stated they did not do so because of public perception and embarrassment.

*‘No, I draw a line at that. The thought of that never appealed to me. I know people that do even now; I’ve seen people in the city centre going through bins outside pubs and at bus stops…. but I’d be too embarrassed to do that!’* (15; male, 53)

All but one participant rolled cigarettes without filters, which was explained by filters being ‘fiddly’ to use when street homeless and by the perception that using filters reduced the strength and ‘purity’ of cigarettes. This was perceived as undesirable because tobacco was a relative luxury and participants wanted to get the most out of each cigarette in order to fulfil their dependency as lastingly as possible.

*‘Well yeah because you smoke tobacco with no filter so it’s stronger, so you don’t need as many. You go back on to fags and it’s like…I need another one!’* (13; male, 33)

### Smoking, quitting and harm reduction: environmental influence, past experience and future needs

Table 2 displays details of participants’ reported current levels of motivation and confidence to quit, showing that, while the majority indicated low motivation, more than a third reported higher levels. One participant with low motivation reported that higher levels of motivation would rely on a solid reason to quit, such as ill health:

*‘If my health really deteriorates, then I would consider…I know I shouldn’t wait until it happens but that’s how I feel at the moment’* (11; male, 44)

**Table 2:** Outline of motivation and confidence to quit scores

Motivation to quit*	
<5	9
>5	6
Motivation to quit**	
<5	7
>5	8

* Scale of 1-10/1=not at all motivated; 10=extremely motivated.

** Scale of 1-10/1=not at all confident; 10=extremely confident.

Most stated that they would feel confident to quit if they attempted to, whereas this tended to correspond with existing plans for quitting in the near future or with successful quitting for a significant period of time previously. Those scoring low confidence levels had either never attempted to quit smoking, or had tried and failed once or numerous times. The psychosocial influence of peers’ smoking behaviours was implied as a reason for low confidence in one participant’s account:

*‘I am not very confident* [that I can quit]*, because I’m always around people that are smoking’* (10; female, 36)

Generally, the influence of homeless peers on smoking behaviour appeared evident, with exceptionally high levels of smoking acknowledged by all participants.

*‘They smoke a hell of a lot more than a normal casual life.’* (15; male, 18)

In terms of other environmental and psychosocial influences, all participants reported to have asked homeless service staff for cigarettes, and the vast majority had either been given or shared a cigarette by or with staff. There was acknowledgement that staff were sometimes reluctant to do this, as it may break the rules of the services, and one participant described the role of personal relationships and privilege in this context:

*‘They’re not allowed to give you cigarettes, but because I get on well with them most of the time, if I say ‘please give me a cigarette’ and they give me one!’* (10; female, 36)

Others stated they were given cigarettes as a reward or acknowledgement for carrying out small jobs around services. One stated that the exchange had broken the ice with staff members and helped him to open up to staff and discuss other issues.

*‘I’ve been in hostels before and I’ve helped out the hostel by tidying the reception area or swept up outside and a member of staff will come outside for a cigarette and offer me one for helping’* (9; male, 40)

The majority of participants had made previous quit attempts and most had plans to reduce their smoking or quit in the future. All of those who had tried to quit in the past had used some harm reduction methods such as nicotine replacement therapies to cut down consumption, and many stated they would be interested in this approach in the future.

*‘I don’t have a date to quit yet but I’d like to be able to quit by reduction and then get off eventually!’* (9; male, 40)

The use of substances other than tobacco was explored and not reported as a concern undermining quitting, except for those who smoked cannabis, as the pathways of administration (usually via cigarette) were interlinked:

*‘It’s very hard to smoke weed on its own. If I was going to give up tobacco, I’d have to give up weed as well so it would be a double effort’* (12; female, 35)

Links between smoking, quitting and other substance use were described in both directions: one participant argued that being drug and alcohol dependent did not deter him from quitting smoking, as he was trying to address all addictions to substances.

*‘I'm looking at all the addictions in my life and using services to get off them....smoking is no different’* (13; male, 33)

Conversely, alcohol use was cited as a reason for relapse into smoking:

Interviewer: ‘Why did you start smoking again?’

*Participant: ‘When I had a drink, I liked a fag and then it just carried on from then’* (9; male 40)

Another participant stated that because he was now abstinent from alcohol, it was helping to limit tobacco consumption.

*‘Since I’ve detoxed off alcohol, I used to smoke 2 or 3 cigs with every can of beer. So now I’m not drinking at all, that cuts all of those ones out!’* (14; male 53)

Quitting smoking was described by some as adding additional stress to already stressful life circumstances. However, some mentioned that frequenting homeless hostels with smoking restrictions in communal areas however was seen as helpful in assisting to reduce cigarette consumption, and several participants residing in hostels commented that it may help them further if smoking restrictions were increased to limit smoking in residents’ rooms, due to the reluctance to going outside for every cigarette:

*‘If I couldn’t smoke in my room, I probably wouldn’t smoke the two or three [cigarettes] I have been smoking, I wouldn’t want to go outside!’* (14; male, 53)

Previous encouragement to address smoking by health professionals was reported by a minority of participants and appeared to consist of reading advertisements in GP surgery or peri-natal service settings, bringing the subject up with the health professional by own initiative, and the professional recommending the local NHS Stop Smoking Services, which had been accessed by 4 participants, none of whom had however managed to quit for a sustained period of time. Relapse was often attributed to exposure to a social environment where smoking was the norm.

A lack of encouragement or active discouragement by health professionals to quit smoking was also detailed by several participants.

*‘I spoke to my GP about me quitting and he said ‘don’t you think you’re trying to do too much with the drinking as well?’ and I am still taking drugs but I was taking them all day, every day and I’ve cut right down, I mean I still use every fortnight and that’s not great but it could be a lot worse and it’s still progress but he said ‘one thing at a time’ but I’m ready and my body is starting to ache and creak and feeling old’* (14; male, 53)

*‘Well, she says to me ‘you’re addressing other things at this time, I don’t think you’re ready to sort this out yet (…). She made me not interested’* (10; female, 36)

Several participants felt that support offers to stop smoking should be more visible and available in a variety of settings accessed by the population, such as high street shop locations and current substance and homeless services. One commented that health professionals and the NHS were not doing enough to address the issue. He stated he attended the GP every fortnight but they had never asked him about smoking:

*‘I’ve seen leaflets stuck on walls but no one has ever said anything and I think they could do a lot more, GPs and in general, the NHS [National Health Service] as a whole, because I think beyond sticking leaflets on walls, they don’t do anywhere near enough’* (14; male, 53)

## Discussion

This study confirmed the high tobacco dependence profile in homeless smokers described previously, as well as levels of motivation and confidence in the ability to quit [17,18] that defies common practice to leave smoking unaddressed in this vulnerable population [11]. It also strongly conveyed the engagement in risk behaviours associated with obtaining tobacco. In addition, the study revealed that participants had often previously engaged in both supported and unsupported quit attempts, indicating a considerable level of initiative, in the light of lacking support of active discouragement by health and homeless service staff. Importantly, addressing smoking was mostly not anticipated to impact negatively on other substance abuse, but was, in some cases, perceived as desirable in the context of addressing other dependencies. Smokefree policies were mentioned by some as helpful in supporting the management of tobacco consumption.

### Study limitations

Limitations of this study include the fact that participants were recruited from a harm reduction service (needle exchange; advice on substance use and sexual health) in the city centre of a UK setting, indicating a level of engagement with health services that may, in other subgroups of the homeless population, be rarer. It is therefore possible that certain parameters, such as motivation/confidence to quit, previous engagement in efforts to give up smoking, and concurrent substance abuse were high compared to the general homeless population, whereas the latter is known to be very high among homeless people [19]. A further limitation of the study is the absence of data from homeless non-smokers who had managed to quit successfully in the past, offering a different perspective on the subjects discussed in the interviews. However, the researchers were not able to identify current non-smokers from their clinical notes, and none of the study participants knew current non-smoking homeless service users that could have been attempted to be recruited using a snowballing strategy. The uneven spread of gender in this study could be viewed as a limitation; however it reflects the majority of males in the general homeless population and is thus unlikely to have unduly skewed the data.

### Lack of acknowledgement of health risks

Findings that participants appeared to know little about the health risks of smoking contrasted results from another study, according to which homeless smokers had high levels of knowledge about the risks of smoking [7]; however, it corresponds to findingsindicating that knowledge of tobacco related harms decreases with decreasing socioeconomic status [20] and is overall not surprising, particularly as a link in the reading ability to the level of tobacco-related knowledge is established [21]. While all participants in this study were able to read and write, homeless people are generally well documented to have lower literacy levels than the general population, with a third of one of the UK’s largest homeless service’s users having difficulty in understanding what they read [22].

A pertinent point was the perceived relative risk of smoking compared to participants’ experience of current life circumstances in general. This mirrored findings from a study on homeless people’s attitude towards death and dying [23], where previous life experiences and losses had reduced perceptions of risk and risk behaviours.

### Lack of encouragement by health and homeless service professionals

Despite smoking being recognised as a significant risk factor for homeless people’s health and most participants in this study (maybe atypically so) seeing their GP or addiction specialists regularly due to concurrent treatment needs related to drug misuse, most had never been encouraged to address smoking. This is in direct contrast to evidence relating to the general population [24], and in opposition to national practice guidelines for health professionals [25], according to which smoking status should be assessed and brief advice to quit be provided at every opportunity, as this is known to be effective in triggering quit attempts and recognised as the most cost effective intervention GPs can engage in.

The reported lack of encouragement or indeed active discouragement to address smoking mirrors findings from other vulnerable populations, such as people with mental illness, where numerous barriers, including staff attitudes and beliefs related to the ‘therapeutic’ effects of smoking on some symptoms of mental illness, potential harms of doing so, and a general ‘first things first’ attitude, marginalising smoking, have been described as part of a complex and intricate smoking culture [26-28]. Similar concerns appear to be of relevance for homeless smokers, who often experience comorbid substance abuse and mental disorder. This is counterintuitive, as smoking cessation does not, as commonly stated, negatively affect outcomes of treatment for other substance misuse, but may indeed enhance them [29]. Several studies indicate that people in treatment for drug and alcohol abuse are often willing and able to quit, albeit with lower long term quit rates than the general population [30], with pilot studies confirming the feasibility and preliminary effectiveness of offering smoking cessation treatment for homeless smokers too [31,32]. It is arguable that practitioners and staff involved in the provision of community services to these vulnerable groups [33] should be trained to offer brief advice and encouragement related to clients’ smoking, with smokefree policies limiting exposure to cues and environmental tobacco smoke.

Encouragement to engage smokers in acceptable harm reduction practices such as using nicotine replacement therapies for cutting down cigarette consumption, should also be practiced by frontline services, and, in the UK, are now in line with new guidelines from the National Institute for Health and Clinical Excellence (NICE) [34].

### High risk smoking behaviours

Our findings of homeless people engaging in potentially risky behaviours related to sourcing and consuming tobacco, such as sharing cigarettes, smoking discarded tobacco and blocking filters, corroborates descriptions from early work in this area [35]. Those participants who reported the use of discarded tobacco stated their reluctance to do so as a means of last resort, with feelings of shame attached. There is no literature available attributing this behaviour to any other population. There are however well documented risk behaviours among other users of addictive substances, such as heroin and crack injectors, where the sharing of injecting equipment is a known, but still common risk behaviour [36].

A notable contrast to findings from an early study is that, while previously a majority of homeless study participants reported to smoke discarded cigarette butts, all of our study participants denied this, stating that even if they picked up discarded butts they always remade them with new cigarette paper. Re-rolling tobacco appeared to be essential for participants and made them feel safer when engaging in what they knew was a risky practice. Concerns over health and transmission of disease in this group were thus apparent. In previous work, transmission risk from smoking discarded tobacco was acknowledged by some participants, identifying herpes and Hepatitis C as transmission risks, whereas transmission of Hepatitis A, influenza and tuberculosis (TB) were more likely [35], especially as TB rates are significant among the homeless [37].

Negotiating how cigarettes were shared appeared to be a complex social interlude around personal preference, negotiating power, the smokers’ relationship and stage of withdrawal. This relationship has not been documented elsewhere but highlights the power and importance of smoking related behaviour in specific social contexts. The use of cigarettes as reward or means of social interaction is well described for mental health settings [28].

### Illicit tobacco use

Cheaper, under the counter rolling tobacco was smoked by most participants, fitting with findings that people buying smuggled tobacco are heavier smokers with higher levels of dependency, living in socially deprived areas and with low educational attainment [38]. Smuggled tobacco has been reported to be viewed positively by low income smokers as a way to combat the smoking’s increasing cost [39], and this was corroborated by this study.

Although participants were aware that, as indicated by earlier studies [40], smoking illicit tobacco was probably worse for them than regulated cigarettes the financial benefits appeared to outweigh the risk of not being able to sustain their dependency without this source. This view was supported by a study of people in disadvantaged communities’ attitudes to contraband cigarettes [31] and another who felt that vendors were doing them a favour, and protecting them from the rising cost of cigarettes [39], further highlighting the vulnerability of smokers from these populations.

## Conclusions

Findings from this study highlight the particular vulnerability of usually heavily dependent homeless smokers, who engage in high risk smoking practices and unique social interplays determined by dependence and need. This and a lack of support or active discouragement to quit by practitioners contrasted the reported levels of motivation and confidence, as well previous initiatives to quit and emphasize the importance of the development of appropriate support strategies for this hard-to-reach group, including at existing service access points, where smokefree policies appear to be a useful means of supporting smokefree environments and behaviours. Such strategies would usefully involve training of staff involved with homeless service users in the community, primary and secondary care; the development of tailored educational information and interventions including harm reduction that could be readily delivered to those motivated to address smoking.

## Competing interests

The authors declare that they have no competing interests.

## Authors’ contributions

LG carried out the research, whereas ER was substantially involved in the conception and design of the study, the development of the coding frame, and in the drafting of the manuscript. Both authors read and approved the final manuscript.

## Pre-publication history

The pre-publication history for this paper can be accessed here:

http://www.biomedcentral.com/1471-2458/13/951/prepub

## References

[B1] Allender S The burden of smoking-related ill health in the UK Tob Control 2009 18 4 262 267 19509003 10.1136/tc.2008.026294

[B2] Gruer L Effect of tobacco smoking on survival of men and women by social position: a 28 year cohort study BMJ 2009 338 b480 19224884 10.1136/bmj.b480 PMC2645845

[B3] Twigg L Moon G Walker S The smoking epidemic in England 2004 London: Health Development Agency

[B4] Jha P Social inequalities in male mortality, and in male mortality from smoking: indirect estimation from national death rates in England and Wales, Poland, and North America Lancet 2006 368 9533 367 370 16876664 10.1016/S0140-6736(06)68975-7

[B5] Royal College of Physicians and Royal College of Psychiatrists Smoking and mental disorder 2013 London: Royal College of Physicians

[B6] Wincup E Buckland G Bayliss R Youth homelessness and substance misuse: report to the drugs and alcohol research unit 2003 London: Home Office, Development and Statistics Directorate

[B7] Butler J Smoking characteristics of a homeless population Subst Abus 2002 23 4 223 231 12438835 10.1080/08897070209511495

[B8] Thompson SJ Risk/protective factors associated with substance use among runaway/homeless youth utilizing emergency shelter services nationwide Subst Abus 2004 25 3 13 26 16150676 10.1300/j465v25n03_02

[B9] Department of Health Healthcare for single homeless people 2010 London: Office of the Chief Analyst, Department of Health

[B10] Hwang SW The health and housing in transition study: a longitudinal study of the health of homeless and vulnerably housed adults in three Canadian cities Int J Public Health 2011 56 6 609 623 21858461 10.1007/s00038-011-0283-3

[B11] Health Development Agency Homelessness, smoking and health 2004 London: Health Development Agency

[B12] Wright NM Tompkins CN How can health services effectively meet the health needs of homeless people? Br J Gen Pract 2006 56 525 286 293 16611519 PMC1832238

[B13] UK, C Homelessness fact file http://www.crisis.org.uk/data/files/publications/factfile_Full.pdf, 2003, Crisis

[B14] Glaser B Strauss A The discovery of grounded theory: strategies for qualitative research 1967 New York: Aldine Publishing Company

[B15] Etter JF Duc TV Perneger TV Validity of the fagerstrom test for nicotine dependence and of the heaviness of smoking index among relatively light smokers Addiction 1999 94 2 269 281 10396794 10.1046/j.1360-0443.1999.94226910.x

[B16] Pope C Ziebland S Mays N Qualitative research in health care. Analysing qualitative data BMJ 2000 320 7227 114 116 10625273 10.1136/bmj.320.7227.114 PMC1117368

[B17] Arnsten JH Smoking behavior and interest in quitting among homeless smokers Addict Behav 2004 29 6 1155 1161 15236817 10.1016/j.addbeh.2004.03.010

[B18] Connor SE Smoking cessation in a homeless population: there is a will, but is there a way? J Gen Intern Med 2002 17 5 369 372 12047734 10.1046/j.1525-1497.2002.10630.x PMC1495046

[B19] Johnson KD Whitbeck LB Hoyt DR Substance abuse disorders among homeless and runaway adolescents J Drug Issues 2005 35 4 799 816 21533015 10.1177/002204260503500407 PMC3083077

[B20] Siahpush M Socioeconomic and country variations in knowledge of health risks of tobacco smoking and toxic constituents of smoke: results from the 2002 international tobacco control (ITC) four country survey Tob Control 2006 15 Suppl 3 iii65 iii70 16754949 10.1136/tc.2005.013276 PMC2593062

[B21] Arnold CL Smoking status, reading level, and knowledge of tobacco effects among low-income pregnant women Prev Med 2001 32 4 313 320 11304092 10.1006/pmed.2000.0815

[B22] Olisa J Patterson J Wright F Homlessness literacy report: turning the key: portraits of low literacy amongst people with experience of homelessness 2010 London: Thames Reach

[B23] Song J Experiences with and attitudes toward death and dying among homeless persons J Gen Intern Med 2007 22 4 427 434 17372788 10.1007/s11606-006-0045-8 PMC1829422

[B24] McEwen A West R Smoking cessation activities by general practitioners and practice nurses Tob Control 2001 10 1 27 32 11226357 10.1136/tc.10.1.27 PMC1763978

[B25] West R McNeill A Raw M Smoking cessation guidelines for health professionals: an update. Health education authority Thorax 2000 55 12 987 999 11083883 10.1136/thorax.55.12.987 PMC1745657

[B26] Parker C McNeill A Ratschen E Tailored tobacco dependence support for mental health patients: a model for inpatient and community services Addiction 2012 107 Suppl 2 18 25 23121356 10.1111/j.1360-0443.2012.04082.x

[B27] Ratschen E Britton J McNeill A The smoking culture in psychiatry: time for change Br J Psychiatry 2011 198 1 6 7 21200069 10.1192/bjp.bp.110.081372

[B28] Lawn SJ Systemic barriers to quitting smoking among institutionalised public mental health service populations: a comparison of two Australian sites Int J Soc Psychiatry 2004 50 3 204 215 15511114 10.1177/0020764004043129

[B29] Kalman D Addressing tobacco use disorder in smokers in early remission from alcohol dependence: the case for integrating smoking cessation services in substance use disorder treatment programs Clin Psychol Rev 2010 30 1 12 24 19748166 10.1016/j.cpr.2009.08.009 PMC2826972

[B30] Baca CT Yahne CE Smoking cessation during substance abuse treatment: what you need to know J Subst Abuse Treat 2009 36 2 205 219 18715746 10.1016/j.jsat.2008.06.003

[B31] Shelley D Smoking cessation among sheltered homeless: a pilot Am J Health Behav 2010 34 5 544 552 20524884 10.5993/ajhb.34.5.4 PMC2882631

[B32] Okuyemi KS Smoking cessation in homeless populations: a pilot clinical trial Nicotine Tob Res 2006 8 5 689 699 17008196 10.1080/14622200600789841

[B33] Bryant J Implementing a smoking cessation program in social and community service organisations: a feasibility and acceptability trial Drug Alcohol Rev 2012 31 5 678 684 22146050 10.1111/j.1465-3362.2011.00391.x

[B34] National Institute for Health and Clinical Excellence Tobacco - harm-reduction approaches to smoking (draft guidance) http://guidance.nice.org.uk/PHG/52, 2012

[B35] Aloot CB Vredevoe DL Brecht ML Evaluation of high-risk smoking practices used by the homeless Cancer Nurs 1993 16 2 123 130 8477400

[B36] Wilkins L Bissell P Meier PS Risky injecting practices associated with snowballing: a qualitative study Drug Alcohol Rev 2010 29 3 256 262 20565517 10.1111/j.1465-3362.2009.00142.x

[B37] Schieffelbein CW Jr Snider DE Jr Tuberculosis control among homeless populations Arch Intern Med 1988 148 8 1843 1846 3401108

[B38] Taylor AJ Langdon M Campion P Smuggled tobacco, deprivation and addiction Eur J Public Health 2005 15 4 399 403 15996974 10.1093/eurpub/cki006

[B39] Wiltshire S "They're doing people a service"-qualitative study of smoking, smuggling, and social deprivation BMJ 2001 323 7306 203 207 11473911 10.1136/bmj.323.7306.203 PMC35272

[B40] Aitken CK Smokers of illicit tobacco report significantly worse health than other smokers Nicotine Tob Res 2009 11 8 996 1001 19541950 10.1093/ntr/ntp102

